# Rho kinase proteins display aberrant upregulation in vascular tumors and contribute to vascular tumor growth

**DOI:** 10.1186/s12885-017-3470-7

**Published:** 2017-07-14

**Authors:** Clarissa N. Amaya, Dianne C. Mitchell, Brad A. Bryan

**Affiliations:** 1 Department of Biomedical Sciences, Texas Tech University Health Sciences Center, Paul L. Foster School of Medicine, Center of Excellence in Cancer Research, 5001 El Paso Drive, MSB1 Room 2111, El Paso, TX 79905 USA; 2 Minerva Genetics, 5130 Gateway Blvd East, Suite 315, El Paso, TX 79905 USA

**Keywords:** Rock, Rho kinase, Angiosarcoma, Hemangioma, Hemangioendothelioma, Hemangiopericytoma, Vascular sarcoma, shRNA

## Abstract

**Background:**

The serine/threonine protein kinases ROCK1 and 2 are key RhoA-mediated regulators of cell shape and cytoskeletal dynamics. These proteins perform multiple functions in vascular endothelial cell physiology and are attractive targets for cancer therapy based on their roles as oncogenes and metastatic promoters. Given their critical functions in both of these processes, we hypothesized that molecular targeting of ROCK proteins would be exceedingly effective against vascular tumors such as hemangiomas and angiosarcomas, which are neoplasms composed of aberrant endothelial cells.

**Methods:**

In this study, we compared ROCK1 and 2 protein expression in a large panel of benign and malignant vascular tumors to that of normal vasculature. We then utilized shRNA technology to knockdown the expression of ROCK1 and 2 in SVR tumor-forming vascular cells, and evaluated tumor size and proliferation rate in a xenograft model. Finally, we employed proteomics and metabolomics to assess how knockdown of the ROCK paralogs induced alterations in protein expression/phosphorylation and metabolite concentrations in the xenograft tumors.

**Results:**

Our findings revealed that ROCK1 was overexpressed in malignant vascular tumors such as hemangioendotheliomas and angiosarcomas, and ROCK2 was overexpressed in both benign and malignant vascular tumors including hemangiomas, hemangioendotheliomas, hemangiopericytomas, and angiosarcomas. shRNA-mediated knockdown of ROCK2, but not ROCK1, in xenograft vascular tumors significantly reduced tumor size and proliferative index compared to control tumors. Proteomics and metabolomics analysis of the xenograft tumors revealed both overlapping as well as unique roles for the ROCK paralogs in regulating signal transduction and metabolite concentrations.

**Conclusions:**

Collectively, these data indicate that ROCK proteins are overexpressed in diverse vascular tumors and suggest that specific targeting of ROCK2 proteins may show efficacy against malignant vascular tumors.

**Electronic supplementary material:**

The online version of this article (doi:10.1186/s12885-017-3470-7) contains supplementary material, which is available to authorized users.

## Background

Vascular tumors are a highly diverse group of aberrant growths which include various benign hemangiomas, borderline malignant hemangioendotheliomas, and malignant hemangiopericytomas and angiosarcomas. Benign vascular tumors display a range of characteristics, from well-defined, non-invasive small vessels to less defined, locally invasive large vessels [1]. These tumors are relatively abundant in the human population, with infantile hemangiomas being the most common tumor in children and cavernous hemangiomas affecting approximately one in every one hundred people. Treatment is not necessary for most benign vascular tumors unless they threaten bodily functions; however radiotherapy and/or embolization have been used with limited success for very large hemangiomas, and beta blockers, which target catecholamine-stimulated beta adrenergic receptor signaling, are considered a highly effective treatment option for pediatric patients with life threatening infantile hemangiomas [1]. In contrast, their malignant vascular tumor counterparts such as angiosarcomas can be highly lethal tumors, and are composed primarily of aberrant lymphatic or vascular endothelial cells [2]. Treatment of angiosarcomas involves radiation, surgery, and neoadjuvant and/or adjuvant chemotherapy with doxorubicin or taxanes, yet the five year survival rate for these patients is abysmally low [3]. Despite their vascular origin, even the addition of novel anti-angiogenic drugs has shown a minimal to absent response in angiosarcoma patients [4], though similar to infantile hemangiomas, beta blockade has recently emerged as a potential therapy against angiosarcomas [5–8]. Effective treatments are desperately needed to increase the progression free survival or overall patient survival in individuals suffering from this highly aggressive sarcoma.

The Rho associated protein kinases (ROCK) 1 and 2 are serine/threonine kinase protein paralogs identified in the 1990’s as direct downstream effectors of Rho-GTPase signaling and are responsible for regulation of the actin cytoskeleton through phosphorylating numerous downstream targets including LIM kinase, myosin regulatory light chain, and the myosin binding subunit of myosin light chain phosphatase [9–11]. Since that time, the ROCK paralogs have been shown to be involved in a variety of cellular processes far beyond regulation of cytoskeletal dynamics, including cell proliferation, apoptosis, and cell differentiation [6]. The role of ROCK proteins in cancer development, progression, and metastasis has been well established in the literature. Regulation of ROCK’s kinase activity is altered in many cancers through modulation of these proteins’ activation processes, altered subcellular localization, and disrupted interactions with regulatory molecules [12]. Elevated protein expression of the ROCK paralogs has been reported across several cancers including hepatocellular carcinoma, osteosarcoma, and breast, colon, and bladder cancers, and the expression of ROCK1 has been shown to have strong prognostic value in colorectal, breast, and bladder cancer [9, 13–17]. Mutations in both ROCK genes have been identified in multiple cancer genomes and some of these mutations result in enhanced kinase activity of the proteins [18–22]. Given their central roles in regulating major oncogenic processes, inhibition of ROCK activity has shown efficacy against tumors in a large number of pre-clinical studies [23–35]. The success of these pre-clinical studies has the potential to translate clinically given that small molecule inhibitors targeting the kinase activity of these proteins are currently in the clinical pipeline against solid tumors, including AT13148 from Astex Pharmaceuticals (currently in Phase I clinical trials). In addition to performing central roles in tumorigenesis, ROCK proteins and their associated signaling pathways have been heavily implicated in regulating angiogenesis, including pathological angiogenesis in a variety of tumors [26, 36–45]. This suggests that not only does inhibition of ROCK activity directly target tumor cell function, but it also limits the blood supply to tumors through disrupting aberrant tumor angiogenesis.

ROCK1 and 2 share a high degree of homology and modulate the activity of many common substrates, however a number of studies have revealed that ROCK1 and 2 additionally play unique and non-overlapping roles in processes such as stress fiber and focal adhesion formation, phagocytosis, apoptosis, inflammation, and multiple aspects of organ and tissue development [46–58]. Our lab has previously used a combination of silencing RNA (shRNA)-mediated gene expression knockdown and a haplo-insufficient animal model to demonstrate that ROCK1 and 2 play unique and overlapping roles in regulating multiple aspects of endothelial function and angiogenesis, with ROCK2 acting as the dominant paralog in normal endothelial cells [39, 42, 59]. More investigations on the individual functions of the ROCK paralogs are needed to elucidate their underlying mechanisms and to determine the predominant paralog in normal and diseased tissues. In the current study, we examined the protein expression patterns of ROCK1 and 2 in a panel of diverse vascular tumors and subsequently employed a shRNA driven approach to elucidate the role of ROCK1 and 2 in a vascular tumor xenograft model.

## Methods

### Immunohistochemistry

Immunohistochemical (IHC) studies were performed on 5 μm thick, formalin fixed, paraffin-embedded sections. These sections were taken from the scrambled control, ROCK1 shRNA, or ROCK2 shRNA xenograft tumors or from a commercially obtained tumor tissue array (US Biomax, Inc.; #SO8010) consisting of 6 cases of angiosarcoma, 2 malignant hemangiopericytomas, 6 borderline malignant hemangioendotheliomas, 6 capillary hemangiomas, 3 granulomatous hemangiomas, 46 cavernous hemangiomas, and 10 normal (aortic or carotid artery) blood vessel tissues. The pathological features of each tumor were confirmed independently by a University Medical Center Pathologist. Sections were deparaffinized, rehydrated, and treated for antigen retrieval using Trilogy (Cell Marque). Nonspecific binding was blocked with background block solution (Cell Marque). Antigens were detected with antibodies purchased from Abcam as follows: ROCK1 (#ab45171), ROCK2 (#ab71598), and Ki67 (#ab15580). Sections were then incubated with the CytoScan Alkaline Phos Detection System (Cell Marque) and detected using the DAB substrate kit (Cell Marque). All slides were counterstained with Hematoxylin. Immunopositivity was quantified blindly using two metrics: the percentage of tissue with positive staining (<25%, 25–50%, 50–75%, or >75%) and the staining intensity (0 = no staining, + = weak staining, ++ = moderate staining, +++ = high staining). IHC scores were determined by multiplying the staining intensity (0 = 0, + = 1, ++ = 2, +++ = 3) by the percent of tissue stained (<25% = 1, 25–50% = 2, 50–75% = 3, >75% = 4) based on previously described methods [60]. For statistical analysis, the Mann-Whitney rank sum test was used. Statistical significance was determined if the two-sided *P* value of the test was <0.05.

### Cell culture and treatment

SVR cells (ATCC; #CRL-2280) were maintained in Dulbecco’s modified Eagle’s media (DMEM) supplemented with 10% fetal bovine serum (FBS), 80 U/ml penicillin, and 50 μg/ml streptomycin C. SVR cells have been used extensively as a model for angiosarcoma given that no reliable human angiosarcoma cell lines are currently capable of forming tumors that recapitulate the human disease [10, 61, 62]. shRNA vectors (SABiosciences) were transfected using Lipofectamine 2000 and cell pools were stably selected using puromycin. The sequences and efficacy of each shRNA and scrambled control vector used in this study have been previously validated by our lab and published [39] (scrambled control: GGAATCTCTCATTCGATGCATAC; ROCK1 shRNA: GCGCAATTGGTAGAAGAATGT; ROCK2 shRNA: AACCAACTGTGAGGCATGTAT). Y-27632 (trans-4-[(1R)-1-aminoethyl]-N-4-pyridinyl-cyclohexanecarboxamide; Santa Cruz Biotechnology) was utilized at 10 μM.

### mRNA expression

For qPCR, total RNA was purified using the Purelink RNA mini kit (Ambion) and converted to cDNA using the Verso cDNA synthesis kit (Thermo-Scientific). qPCR was performed in triplicate using SYBR Green-based probes against ROCK1 (SABiosciences; #PPM04660B), ROCK2 (SABiosciences; #PPM36940C), or GAPDH (SABiosciences; #PPM02946E). Assays were run on an ABI7900HT real time PCR system (Applied Biosystems).

### Angiosarcoma xenograft model

All xenograft experiments were approved by and performed in accordance to Texas Tech University Health Sciences Center Institutional Animal Care and Use Committee (IACUC) regulations for the care and use of animals in experimental procedures (IACUC protocol # 11035), and all efforts were made to minimize suffering. Animals were housed 4 per cage in a temperature-controlled animal facility on a 12 h–12 h light-dark cycle. Animals had free access to chow and water. Xenograft angiosarcoma tumors were generated by subcutaneous injection of 1 × 105 SVR cells (scrambled control, ROCK1 shRNA, or ROCK2 shRNA) into the dorsolateral flanks of 4 week old female mice as previously described [63, 64]. Body weight and tumor volume of the animals were measured once a week to ensure health of the animals. The mice were observed daily for ulceration, abdominal swelling, emaciation and/or other signs of distress, and tumor burden did not interfere with the ability of the mice to move freely. When the scrambled control tumors reached approximately 1 cm in diameter, the mice were sacrificed by CO2 asphyxiation followed by cervical dislocation, and the tumors from all treatment groups were collected and weighed. Statistical significance in tumor weight was determined using an unpaired two-tailed t-test with Graphpad Prism version 6.05.

### Omics analysis

Hybridization and analysis of the high throughput antibody arrays were performed on tumor lysates using the Phospho-Explorer Antibody Array contract service offered by Full Moon Biosystems (Sunnyvale, CA). ^1^H NMR analysis was performed on tumor lysates using the contract service offered by Chenomx Inc. (Edmonton, Canada). Normalized heatmap data was generated in Cluster 3.0 software (http://bonsai.hgc.jp/~mdehoon/software/cluster/software.htm) using unsupervised hierarchical clustering analysis with an uncentered correlation similarity metric and centroid linkage. Heatmaps were visualized using Java Treeview software (http://jtreeview.sourceforge.net/). Physical and functional associations of the omics data were performed using Metacore Pathway Analysis Software (Thompson Reuters, New York City, NY). For both the proteomics and metabolomics analysis, independent biological samples were tested in triplicate.

### Western blot analysis

Protein lysates from the xenograft tumors were subjected to SDS-PAGE and transferred to PVDF membrane. Membranes were blocked using 3% bovine serum albumin and probed with the following antibodies as indicated: p53 (Cell Signaling #2524), Chk1 (Cell Signaling #2360), Fadd (Abcam #ab24533), Nfkb-p105/p50 (pSer337) (ThermoFisher #PA5–37658), Casp6 (Cell Signaling #9762), Nfkb-p65 (pSer536) (Cell Signaling #3033), and actin (Santa Cruz Biotechnology #sc7319). Appropriate secondary antibodies and chemiluminescent detection substrate was used for imaging of the bands.

## Results

### ROCK protein expression is elevated in vascular tumors

**Fig. 1:**
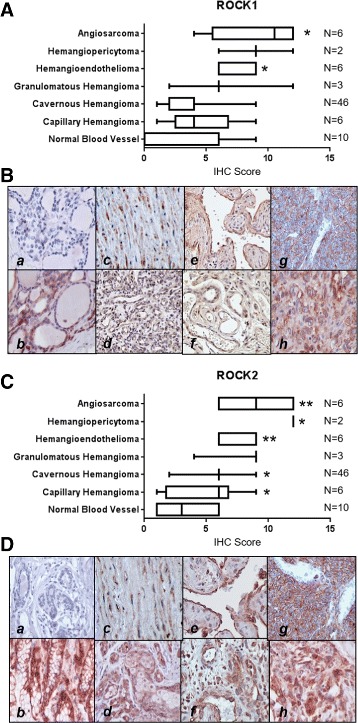
ROCK1 and ROCK2 are overexpressed in vascular tumors. **a** Box and whisker plots indicating the mean immunohistochemical staining score for ROCK1 in a panel of human tissues obtained from normal endothelium and benign, borderline, and malignant vascular tumors. The number of tumor samples tested (N) for each tissue type is indicated to the right of the plot. For statistical analysis, the Mann-Whitney rank sum test was used. Statistical significance was determined if the two-sided *P* value of the test was <0.05. **b** Representative 600× images of immunohistochemical staining for ROCK1 in a panel of human tissues obtained from normal endothelium and benign, borderline, and malignant vascular tumors (*a =* negative control*, b =* positive control*, c =* normal endothelium*, d =* capillary hemangioma*, e =* cavernous hemangioma*, f =* granulomatous hemangioma*, g =* hemangiopericytoma*, h =* angiosarcoma). Negative controls lacking the primary antibody and positive controls from the kidney were used to ensure immunopositivity was reliable. Brown staining indicates immunopositivity. **c** Box and whisker plots indicating the mean immunohistochemical staining score for ROCK2 in a panel of human tissues obtained from normal endothelium and benign, borderline, and malignant vascular tumors. The number of tumor samples tested (N) for each tissue type is indicated to the right of the plot. For statistical analysis, the Mann-Whitney rank sum test was used. Statistical significance was determined if the two-sided *P* value of the test was <0.05. **d** Representative 600× images of immunohistochemical staining for ROCK2 in a panel of human tissues obtained from normal endothelium and benign, borderline, and malignant vascular tumors (*a =* negative control*, b =* positive control*, c =* normal endothelium*, d =* capillary hemangioma*, e =* cavernous hemangioma*, f =* granulomatous hemangioma*, g =* hemangiopericytoma*, h =* angiosarcoma). Negative controls lacking the primary antibody and positive controls from the kidney were used to ensure immunopositivity was reliable. Brown staining indicates immunopositivity. Please see Additional file 3 for high resolution image

ROCK1 and 2 have been shown to be ubiquitously expressed throughout the body, with preferentially higher levels of ROCK2 found in the muscle and brain [11, 65, 66]. Several studies have indicated that ROCK protein expression is elevated in some carcinomas [14, 15, 17, 67, 68], yet it is unknown if these proteins are similarly overexpressed in vascular tumors, particularly given their central role in normal and aberrant vascular function. To address this, we performed immunohistochemistry to compare ROCK1 and 2 expression in normal vasculature, as well as benign, borderline, and malignant vascular tumors. The clinicopathological features of patients associated with this panel of tumors is depicted in Table 1. Based on the staining performed, the expression of ROCK1 is significantly elevated in hemangioendotheliomas and angiosarcomas relative to normal endothelium (Fig. 1a & b). ROCK2 protein is significantly increased in benign vascular tumors including capillary and cavernous hemangiomas, as well as malignant hemangioendotheliomas, hemangiopericytomas, and angiosarcomas (Fig. 1c & d). This suggests that ROCK1 and 2 proteins are upregulated across a broad range of vascular tumors and may serve as excellent therapeutic targets against these tumor types.

**Table 1:** Vascular tumor and control patient characteristics

Variable	Overall	Malignant	Borderline	Benign	Normal
# patient samples	80	8	6	56	10
Age [mean years (s.d.)]	41 ± 17	53 ± 19	36 ± 15	40 ± 17	34 ± 14
Age [median years (range)]	42 (80)	53 (64)	35 (44)	42 (71)	32 (44)
Sex	42F, 39 M	4F, 4 M	6F, 0 M	27F, 29 M	5F, 5 M
Location (# tissue samples)					
Artery	15	1	2	2	10
Cerebrum	2	0	0	2	0
Fallopian Tube	1	1	0	0	0
Fibrous Tissue	1	1	0	0	0
Heart	2	1	1	0	0
Ligament	1	0	1	0	0
Liver	36	1	0	35	0
Lung	1	1	0	0	0
Mesentery	1	0	0	1	0
Skin	12	1	2	9	0
Spleen	4	1	0	3	0
Thyroid	1	0	0	1	0
Tongue	2	0	0	2	0
Vulva	1	0	0	1	0

*M* male, *F* female

### shRNA mediated knockdown of ROCK1 and 2 in a malignant vascular tumor cell line

**Fig. 2:**
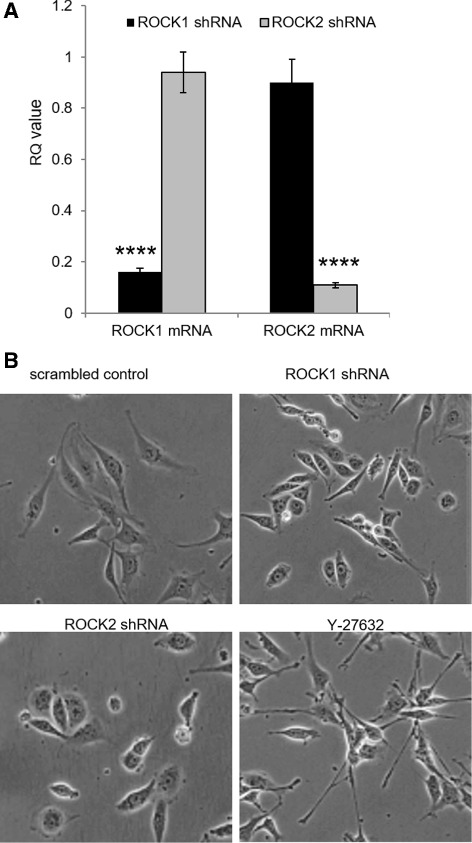
shRNA-mediated knockdown of ROCK1 & 2 in SVR cells. **a** qPCR quantification of the levels of ROCK1 and 2 mRNA in SVR cells harboring shRNA vectors for scrambled control, ROCK1, or ROCK2. GAPDH mRNA was used as a normalization control for RQ calculations. For statistical analysis, the Student’s t-test was used. Statistical significance was determined if the two-sided *P* value of the test was <0.05. **b** Images were collected at 400× total magnification for SVR cells harboring shRNA vectors for scrambled control, ROCK1, or ROCK2 or treated with the pharmacological inhibitor Y-27632 (24 h; 10 μM). Please see Additional file 4 for high resolution image

Given our data demonstrating that ROCK1 and 2 are expressed across benign, borderline, and malignant vascular tumors, we sought to test if reducing the activity of the ROCK proteins would disrupt vascular tumor progression in a xenograft tumor model. To accomplish this, we stably knocked down the expression of ROCK1 and 2 in SVR cells, an established tumorigenic vascular cell line, using shRNA vectors previously reported by our lab [39]. Validation of the knockdowns at the mRNA level is shown in Fig. 2a. While knockdown of ROCK1 or 2 did not alter the proliferation rate of the SVR cells (*data not shown*), changes in cellular morphology were observed in ROCK2 knockdown cells compared to controls, whereby the ROCK2 knockdowns exhibited a more round, less extended appearance (Fig. 2b). While little/no change was observed in ROCK1 knockdowns, addition of the ROCK1 and 2 pharmacological inhibitor Y-27632 resulted in very unique morphological changes including neurite-like extensions protruding from the cell bodies as have been previously reported for other cell types [69] (Fig. 2b).

### shRNA mediated knockdown of ROCK2 inhibits the growth of xenograft vascular tumors

**Fig. 3:**
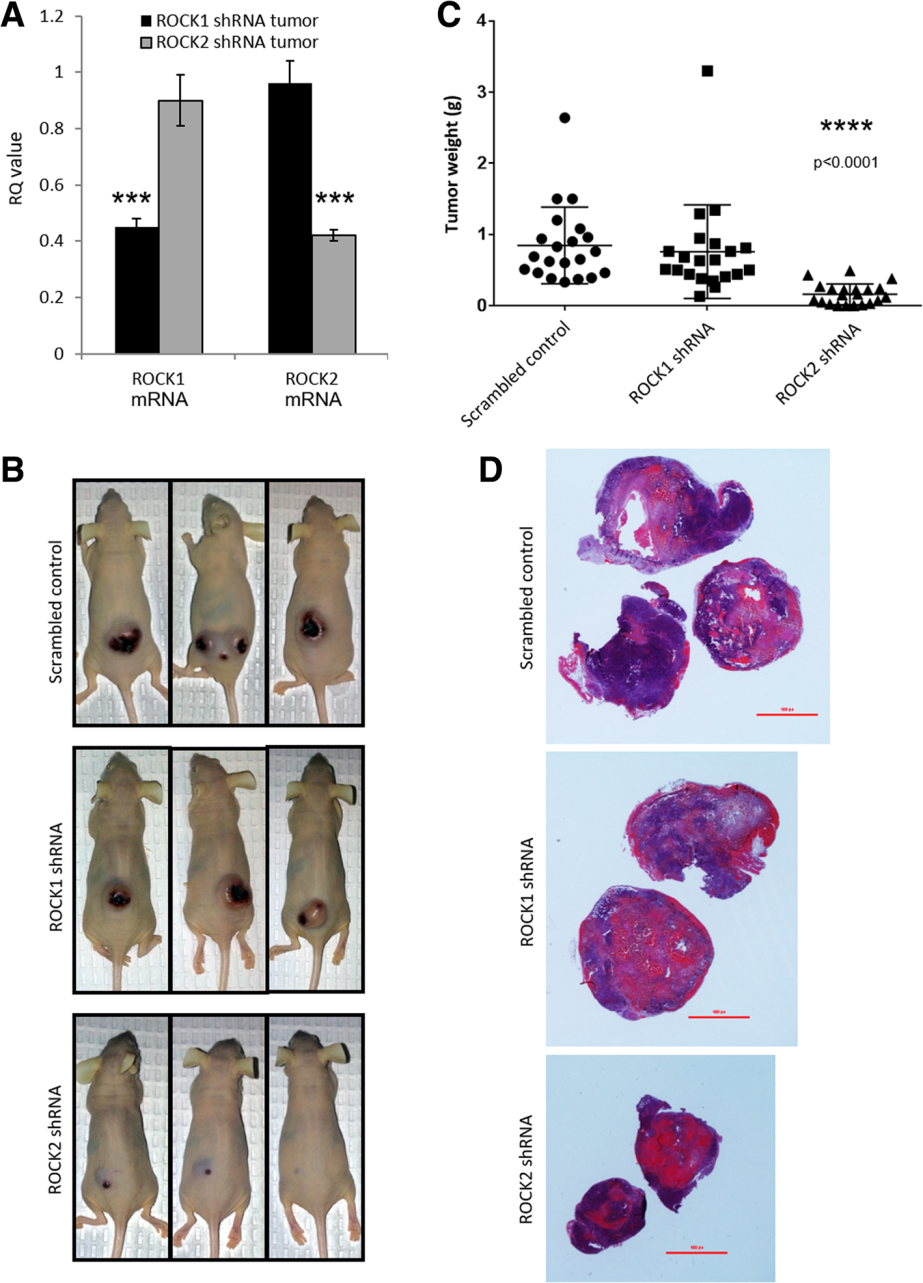
ROCK1 & 2 knockdowns in a xenograft vascular tumor model. **a** qPCR quantification of the levels of ROCK1 and ROCK2 mRNA in the SVR xenograft tumors relative to the scrambled control. GAPDH mRNA was used as a normalization control for RQ calculations. For statistical analysis, the Student’s t-test was used. Statistical significance was determined if the two-sided *P* value of the test was <0.05. **b** Representative images of mice harboring subcutaneous scrambled control, ROCK1 shRNA, and ROCK2 shRNA vascular xenograft tumors. **c** Box and whisker plot showing the distribution of tumor weights in scrambled control, ROCK1 shRNA and ROCK2 shRNA vascular xenograft tumors. Asterisks indicate *p* value <0.0005. (**d**) Low magnification H&E staining of whole tumor sections showing relative tumor sizes following removal from the host. Please see Additional file 5 for high resolution image

We have previously shown that pharmacological inhibition of both ROCK1 and 2 with Y-27632 reduces SVR tumor size in a xenograft model [39]. To evaluate the contribution of each ROCK protein to vascular tumor growth, scrambled control, ROCK1 or 2 knockdown SVR tumor cells were injected into the dorso-lateral flanks of nude mice (*N* = 21 mice per condition) and allowed to grow until the scrambled control tumors reached approximately 1 cm^3^ (approximately 3 weeks post-injection). Knockdown of ROCK1 and 2 in the tumors was confirmed via qPCR (Fig. 3a). At the time of harvesting, the tumors on the mice were photographed (Fig. 3b) and subsequently harvested and weighed (Fig. 3c). No difference in tumor size or weight was observed between the ROCK1 knockdown tumors and the scrambled control tumors (control = 0.85 ± 0.12 g/tumor; ROCK1 knockdown = 0.76 ± 0.14; *p* = 0.64), however ROCK2 knockdown tumors weighed significantly less than the scrambled control tumors (control = 0.85 ± 0.12 g/tumor; ROCK2 knockdown = 0.16 ± 0.03 g/tumor; *p* < 0.0001). The scrambled control and ROCK1 shRNA xenograft tumor-bearing mice were largely ulcerated and openly hemorrhaging on the primary lesion, however ROCK2 shRNA xenograft tumors exhibited no significant dermatological ulcerations. Low magnification H&E staining of representative whole tumor sections is shown in Fig. 3d.

**Fig. 4:**
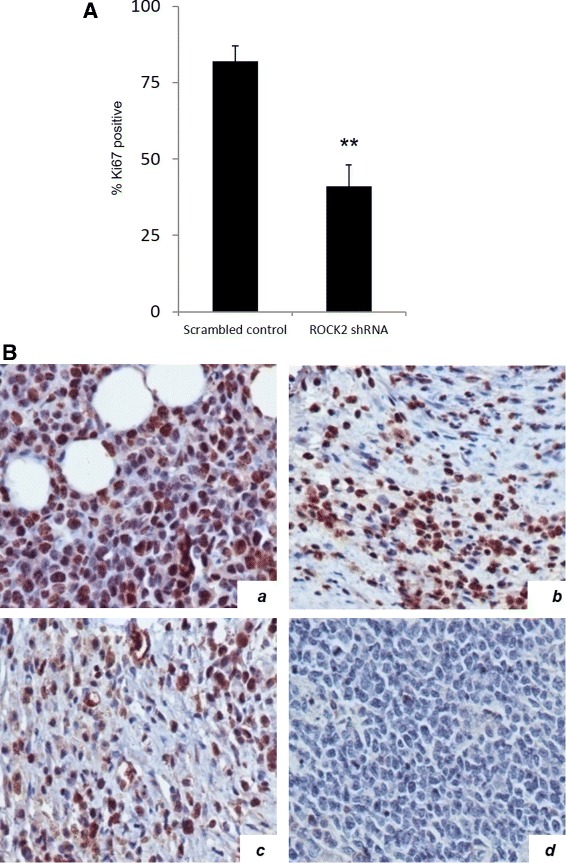
ROCK2 knockdown results in reduced proliferation in a xenograft vascular tumor model. **a** Histogram depicting immunopositivity for Ki67 in scrambled control and ROCK2 knockdown xenograft vascular tumors. Data presented is the mean plus or minus the standard deviation. Asterisks indicate *p* value <0.005. **b** Representative images of scrambled control (*a*) and ROCK2 knockdown (*b*) angiosarcoma tumors stained via immunohistochemistry for the proliferative marker Ki67 (immunopositivity is brown). Positive control (*c*) is Ki67 staining of a breast carcinoma and negative control (*d*) is the xenograft vascular tumor minus primary antibody incubation. Please see Additional file 6 for high resolution image

We analyzed the proliferation rates of scrambled control and ROCK2 shRNA knockdown xenograft angiosarcoma tumors using immunohistochemical staining for the proliferative marker Ki67. ROCK2 knockdown tumors exhibited a significantly reduced Ki67 index compared to the scrambled control (Fig. 4a & b), thus corroborating the effects on tumor size displayed in the ROCK2 knockdown tumors.

### Molecular characterization of xenograft vascular tumors deficient in ROCK1 or 2

**Fig. 5:**
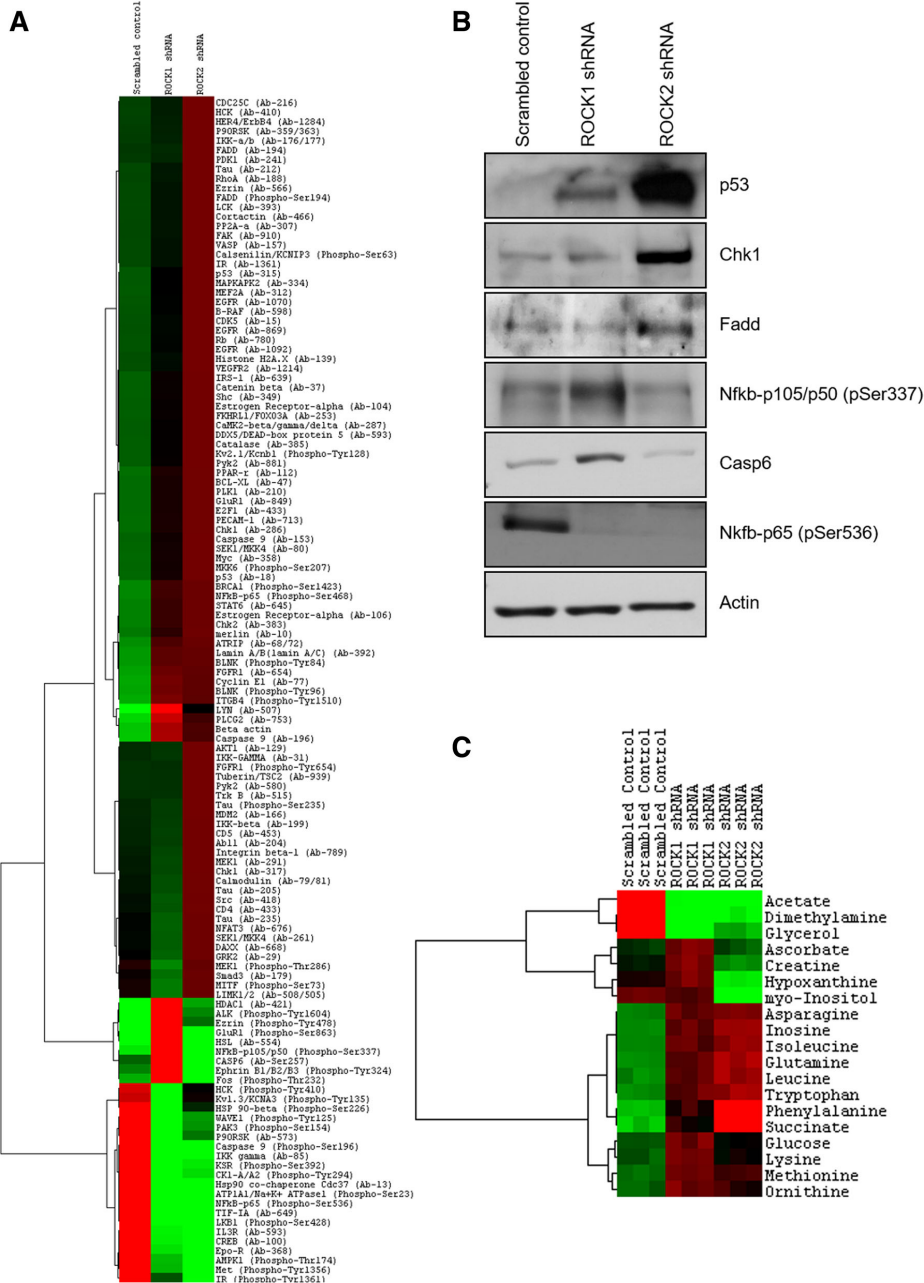
Omics characterization of ROCK1 and 2 shRNA knockdown SVR cells. **a** Lysates from SVR tumors harboring shRNA vectors for scrambled control, ROCK1, or ROCK2 were subjected to Full Moon Phospho Explorer Antibody arrays. Two-fold or more protein expression/modification changes between the knockdown and the control tumors are depicted via heatmap analysis. Each lane is the mean of three independent biological replicates. (*red = upregulated; green = downregulated*) **b** Western blot analysis detecting the expression of key cell cycle/survival regulators in protein extracts collected from scrambled control and ROCK2 knockdown xenograft vascular tumors. **c** Lysates from SVR tumors harboring shRNA vectors for scrambled control, ROCK1, or ROCK2 were subjected to ^1^H NMR analysis metabolomics analysis. Two-fold or more metabolite concentration changes between the knockdown and the control cells are depicted via heatmap analysis. Each column represents the triplicate mean of an individual biological replicate (*red = upregulated; green = downregulated*). Please see Additional file 7 for high resolution image

To characterize the molecular phenotype of ROCK1 and 2 knockdown SVR xenograft tumors, we performed antibody array and metabolomics-based analysis on tumors harvested from the host mice. Using antibody arrays that quantified 1318 site-specific and phospho-specific protein targets from over 30 cellular signaling pathways, we identified 125 proteins whose expression/modifications were up- or down-regulated in the ROCK1 or 2 knockdown cells by over 2-fold relative to the scrambled control tumors (Fig. 5a). The normalized intensity values for all protein expression/modification changes identified via the antibody array can be found in Additional file 1. ROCK1 and 2 have been shown previously to perform both overlapping as well as non-overlapping roles in endothelial cells [39]. The majority of proteins and their modifications were similarly expressed between ROCK1 and 2 shRNA knockout tumors relative to the control tumors, and based on Metacore network analysis these proteins were overrepresented in pathways involved in development/hematopoiesis, inflammation/immune response, and cell cycle regulation. We additionally observed non-overlapping changes in protein expression/modification in the ROCK1 and 2 shRNA knockdown SVR tumors, and based on Metacore network analysis these changes were reflected in proteins involved in immune response/inflammation and cell cycle/survival regulation. Using Western blot analysis, we confirmed a subset of the data obtained in our antibody array by analyzing tumor lysates collected from ROCK1 and 2 shRNA knockdowns and scrambled control xenograft angiosarcoma tumors, revealing ROCK1 shRNA mediated increases in p53, Nfkb-p105/p50 (pSer337), and Casp6 levels (Fig. 5b). ROCK2 shRNA mediated increases in p53, Fadd, and Chk1, while Nfkb-p65 (pSer537) was decreased in both knockdowns compared to the scrambled control.

As metabolic differences have been observed between cancer and normal cells, and many of these metabolic pathways are potential therapeutic targets [70], we sought to evaluate the contribution of ROCK signaling to vascular tumor cell metabolism. ^1^H NMR analysis of tumor lysates revealed two-fold or higher alterations in the levels of 19 metabolites between the ROCK knockdown and scrambled control tumors (Fig. 5c). ROCK1 and 2 knockdown cells displayed similar metabolic profiles for approximately 80% of the metabolites, with differential concentrations of metabolites occurring for ascorbate, creatine, hypoxanthine, and myo-inositol. The normalized data for all intracellular metabolite concentrations can be found in Additional file 2.

## Discussion

Previous publications from our lab and others have provided evidence that modulation of cell shape and cytoskeletal dynamics plays a major role in regulating key endothelial processes. For instance, manipulation of endothelial cell shape and actin organization results in gene expression alterations of approximately 8% of the global genome potentially through altering chromosomal boundaries within the nucleus [71, 72]. Specific disruption of the activity of the cell shape regulators RhoA and ROCK in endothelial cells blocks a number of developmental and cellular properties such as angiogenesis, vascular formation during embryogenesis, and lung capillary development [39, 42, 73, 74]. In addition to altering physiological vascular properties, inhibition of ROCK activity leads to anti-angiogenic effects on the capillary networks of gliomas and prostate adenocarcinomas [41, 43], suggesting an effective role for ROCK inhibition as an anti-angiogenic agent against solid tumors. Furthermore, the ROCK proteins play a prominent role in the proliferation, invasion, and metastasis of tumor cells through modulating cytoskeletal dynamics and other cellular processes [27], and a wealth of preclinical studies have demonstrated the efficacy of ROCK inhibition in the treatment of a variety of cancers over the last 1.5 decades [75].

ROCK1 and ROCK2 are ubiquitously expressed across tissues from early embryonic development to adulthood, though preferential expression of these proteins has been observed in some tissues [11, 66, 76]. Our data revealed that both ROCK1 and 2 protein levels were elevated across vascular tumors relative to normal endothelium. Indeed, ROCK proteins have been found to be aberrantly increased in a variety of more common carcinomas [14, 15, 17, 64, 68]. We suspect that overexpression of ROCK proteins in benign and malignant vascular tumors is a key process whereby these tumors hijack normal cytoskeletal processes to increase invasive and metastatic cell behavior, and therefore may be a selectively preferable therapeutic target whose disruption could prove beneficial to enhancing patient treatment. Thus we hypothesized that targeting ROCK activity may show efficacy against vascular tumors. ROCK1 and 2 shRNA xenograft tumors displayed overlapping and unique roles in both protein expression/modification as well as metabolite concentrations. It has been reported that ROCK proteins display overlapping and unique roles [39, 46–58], and a handful of reports have implicated ROCK proteins in the regulation of metabolism, particularly regarding insulin resistance and glucose metabolism [77, 78]. Further studies are necessary to identify the unique and overlapping roles of ROCK proteins in these particular metabolic processes, and our data suggests that similar to previously reported paralog-specific transcriptional changes [39] and unique protein expression changes reported in the current study, metabolic targets may be differentially regulated by the ROCK proteins as well. Our animal studies revealed that knockdown of ROCK2, but not ROCK1, greatly reduced xenograft tumor volume in an established xenograft vascular tumor model. We suspect the observed reduction in tumor growth in the ROCK2 knockdown tumors is due, in part, to paralog-specific regulation of cell cycle, survival, and checkpoint modulators that contribute to central processes previously shown to be regulated extensively by the ROCK proteins [77].

## Conclusions

ROCK inhibition has strong promise for effective translation into the clinic for the treatment of angiosarcomas and other solid tumors. Indeed, ROCK inhibitors are currently in use or in clinical trials for a variety of diseases including cerebral vasospasm after subarachnoid hemorrhage, hypertension, atherosclerosis, and aortic stiffness [78]. Though no ROCK inhibitors are currently approved for clinical use in the treatment of cancers, the ROCK inhibitor AT13148 is currently in phase I clinical trials, and several other small molecule inhibitors against ROCK proteins have shown efficacy against carcinomas in preclinical tumor models [27, 79–81]. While several overlapping roles have been identified for ROCK proteins, interest in selectively targeting each of the ROCK paralogs has recently gained popularity due to the unique roles that are reported in the literature regarding these proteins [6]. Thus, a strategy that utilizes specific targeting of ROCK paralogs in a context dependent manner could lead to efficiency in achieving optimal anti-cancer results in the clinic. Drugs such as the potent selective inhibitor of ROCK2, Slx-2119 [82, 83], may pave the way for future selective inhibition of ROCK-specific paralogs to achieve optimized therapeutic efficacy.

## Additional files

Additional file 1: Analysis of global protein phosphorylation in ROCK1 and 2 knockdown SVR cells. A high throughput antibody array composed of 1358 antibodies covering more than 20 central signaling pathways was performed on lysates collected from ROCK1 and 2 shRNA SVR cells and a corresponding scrambled shRNA control. The data is presented as the normalized median signal values of each antibody spot. Moreover, the fold expression change was compared between ROCK shRNA and scrambled shRNA control SVR cells. (XLS 241 kb)

Additional file 2: Analysis of intracellular metabolite concentrates in ROCK1 and 2 knockdown SVR cells. Triplicate values for metabolite concentrations (μM) detected by H_1_ NMR analysis in ROCK shRNA and scrambled shRNA control SVR cells. (XLS 39 kb)

Additional file 3: High resolution image of Figure 1. (PNG 1490 kb)

Additional file 4: High resolution image of Figure 2. (PNG 611 kb)

Additional file 5: High resolution image of Figure 3. (PNG 1350 kb)

Additional file 6: High resolution image of Figure 4. (PNG 1820 kb)

Additional file 7: High resolution image of Figure 5. (PNG 477 kb)

## Abbreviations

cDNA: Complementary DNA
DAB: 3,3′-diaminobenzidine tetrahydrochloride
DMEM: Dulbecco’s Modified Eagle Medium
FBS: Fetal bovine serum
GTPase: Guanosine triphosphate-ase
H&E: Hematoxylin and eosin
IACUC: Institutional animal care and use committee
IHC: Immunohistochemical
NMR: Nuclear magnetic resonance
qPCR: Quantitative polymerase chain reaction
Rho: Rho GTPase binding protein
RNA: Ribonucleic acid
ROCK: Rho associated, coiled-coil-containing protein kinase
shRNA: Small hairpin ribonucleic acid
