# Supplementary figures and images for: Rho kinase proteins display aberrant upregulation in vascular tumors and contribute to vascular tumor growth
Source: BMC Cancer. 2017 Jul 14;17:485. doi: 10.1186/s12885-017-3470-7 (PMC5513090; doi:10.1186/s12885-017-3470-7)

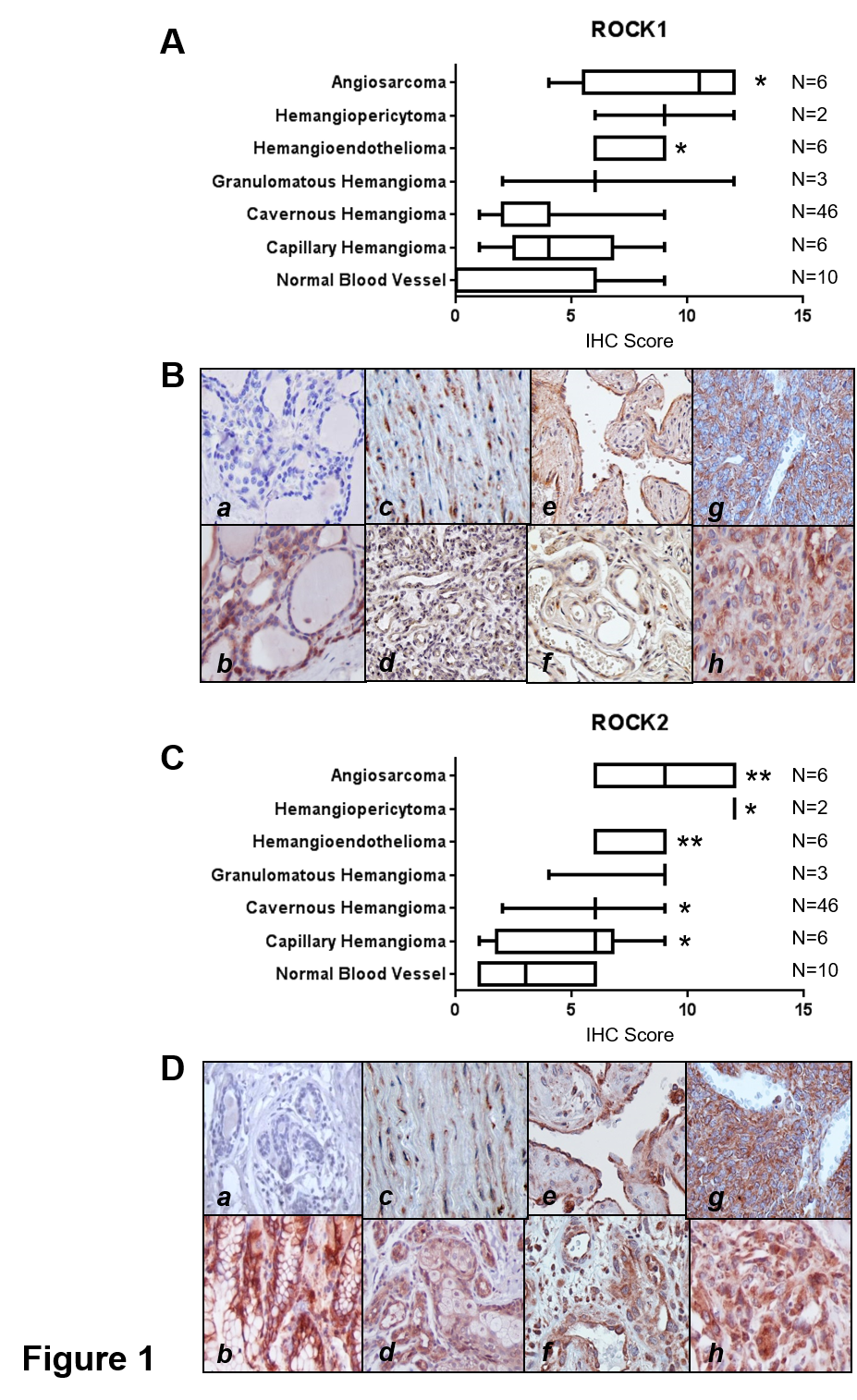

Supplement: Supplementary file 3 — High resolution image of Figure 1. (PNG 1490 kb) [file 12885_2017_3470_MOESM3_ESM.png]

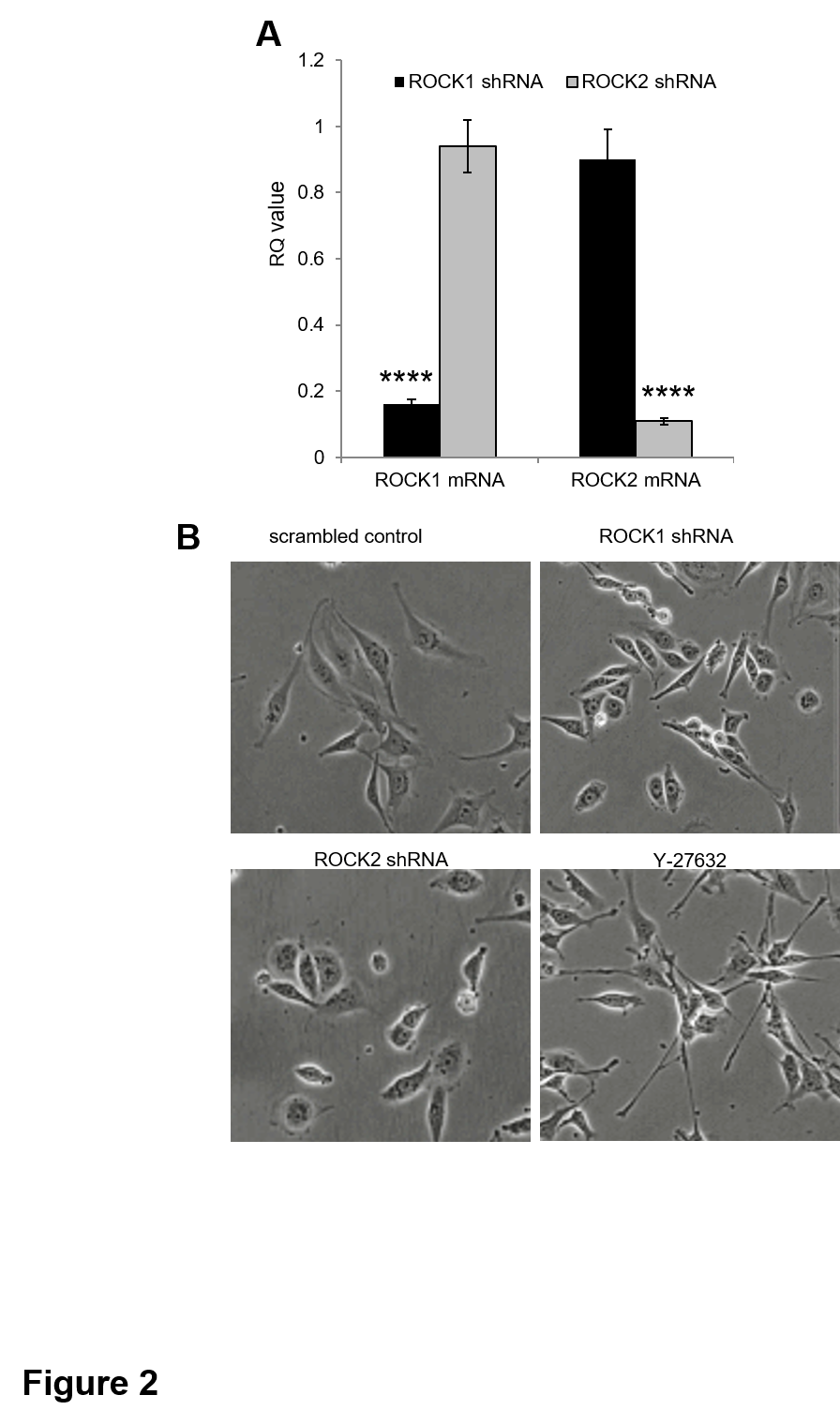

Supplement: Supplementary file 4 — High resolution image of Figure 2. (PNG 611 kb) [file 12885_2017_3470_MOESM4_ESM.png]

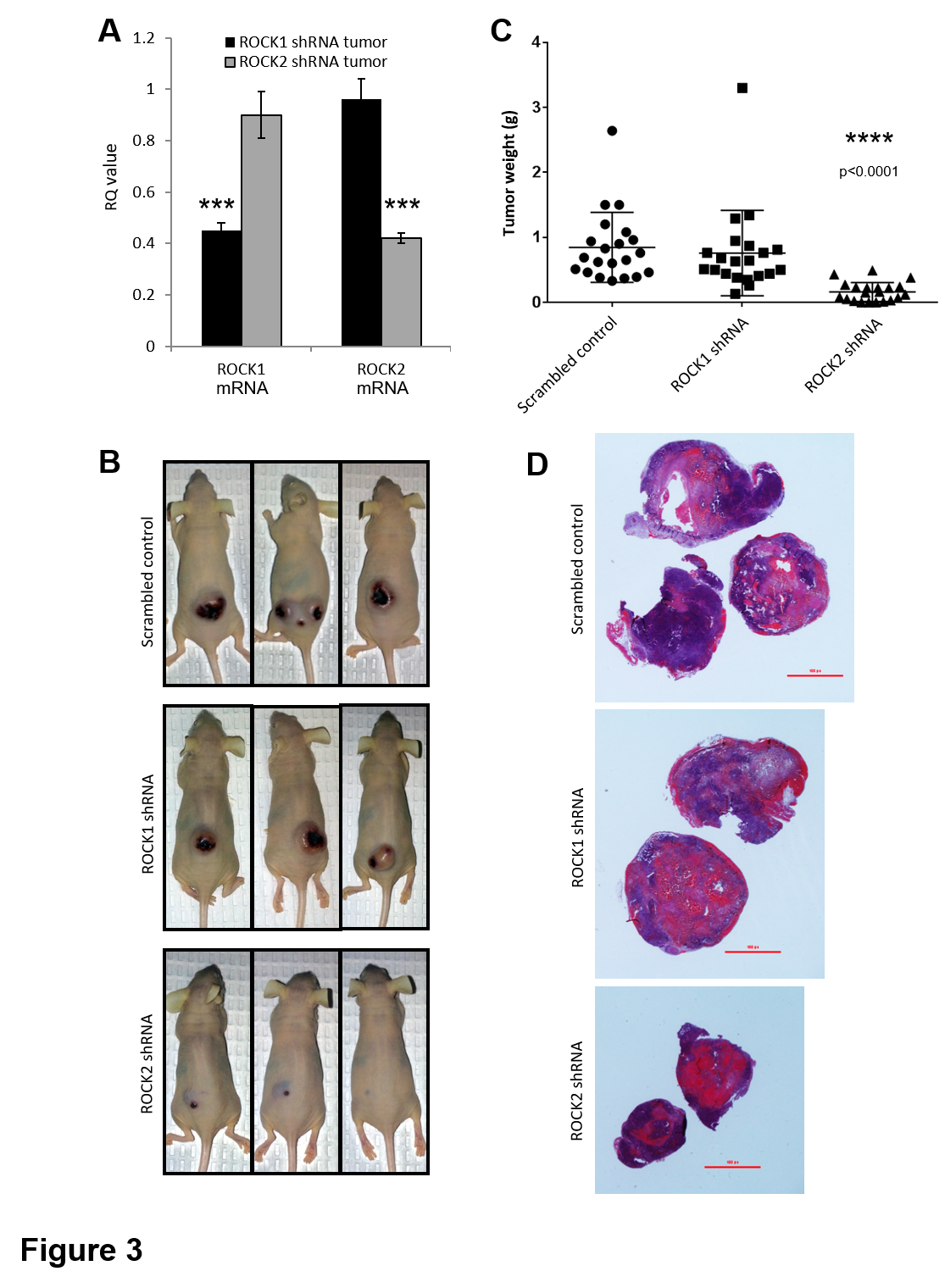

Supplement: Supplementary file 5 — High resolution image of Figure 3. (PNG 1350 kb) [file 12885_2017_3470_MOESM5_ESM.png]

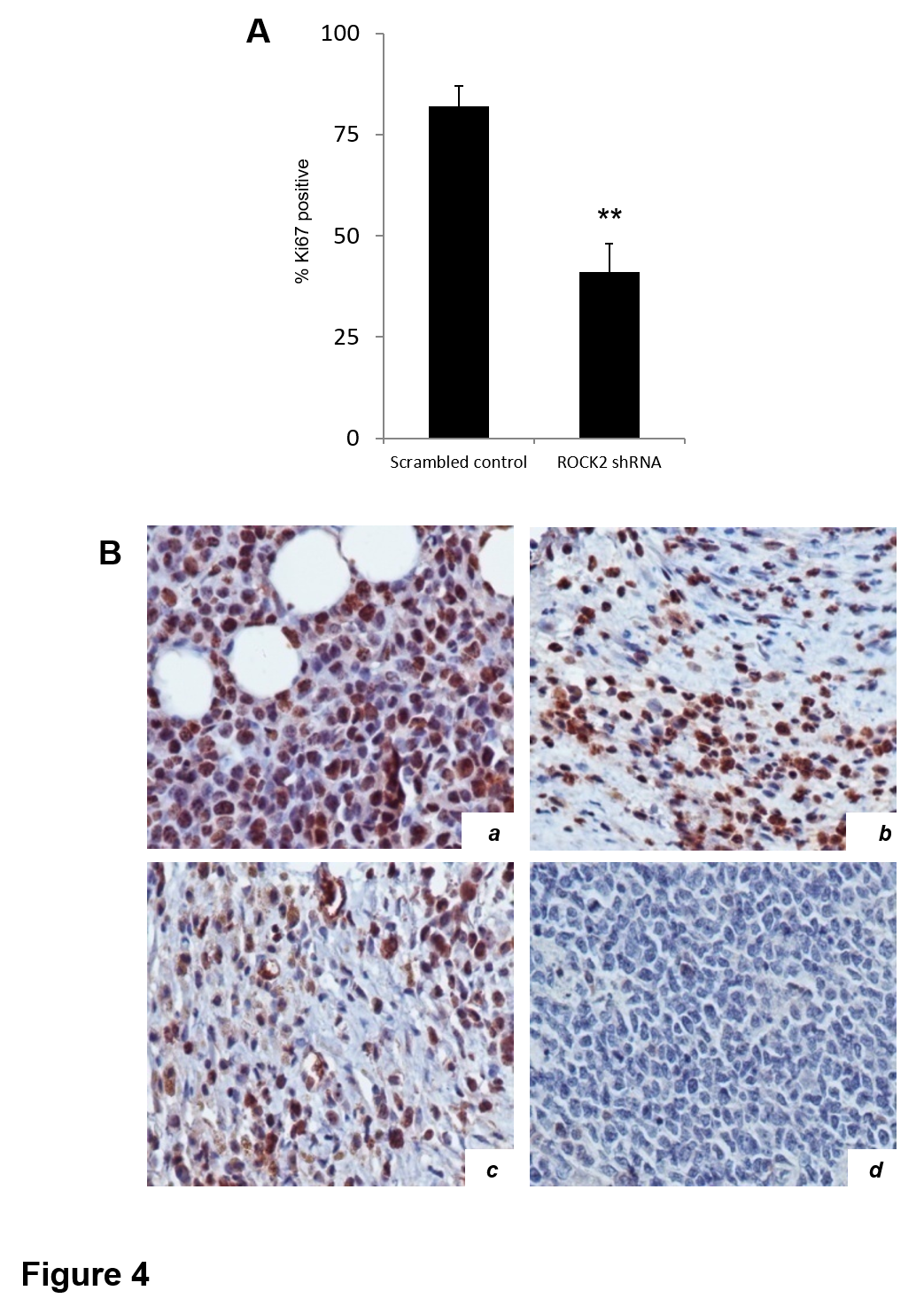

Supplement: Supplementary file 6 — High resolution image of Figure 4. (PNG 1820 kb) [file 12885_2017_3470_MOESM6_ESM.png]

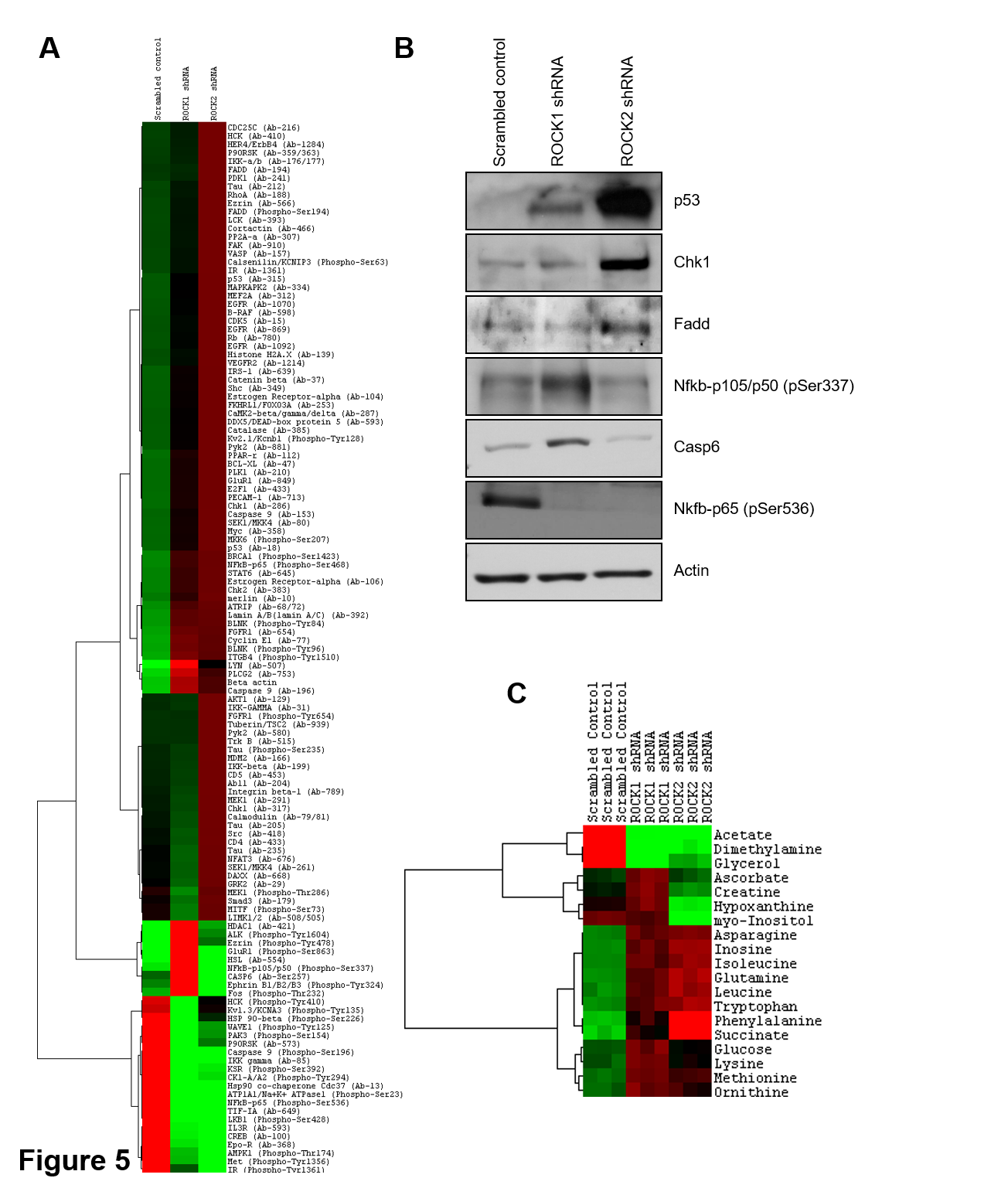

Supplement: Supplementary file 7 — High resolution image of Figure 5. (PNG 477 kb) [file 12885_2017_3470_MOESM7_ESM.png]
